# Disabled people in the time of COVID-19: identifying needs, promoting inclusivity

**DOI:** 10.7189/jogh.11.03007

**Published:** 2021-01-16

**Authors:** Elena S Rotarou, Dikaios Sakellariou, Emily J Kakoullis, Narelle Warren

**Affiliations:** 1 Faculty of Medicine and Science, Universidad San Sebastián, Chile; 2 School of Healthcare Sciences, Cardiff University, Cardiff, UK; 3 School of Law and Politics, Cardiff University, Cardiff, UK; 4 School of Social Sciences, Monash University, Australia

The COVID-19 pandemic has brought to stark relief, and further exacerbated, social disparities, including those experienced by disabled people, a global population of over 1 billion people, according to 2010 global population estimates [1]. Disabled people experience entrenched structural disadvantage, including barriers to accessing health care, increased poverty, lower employment, and lower education levels, in comparison to the general population [1].

It is important that the needs of disabled people are not ignored during the pandemic [2]. The COVID-19 pandemic, and the government measures taken to address it, intersect with existing inequalities to create cascades of disadvantage across multiple domains, including health care, education, and employment. Government responses impact on disabled people’s ability to protect against the pandemic, to mitigate the effects of social distancing measures, to buffer against the ensuing financial downturn, and to navigate the disruption in the provision of general and specialist health services, including in their access to COVID-19-related health services [3,4].

During the pandemic, ‘disablism’, defined as those discourses and practices that exclude, discriminate against, and oppress disabled people– has put disabled people at risk, pointing to a ‘disvaluing’ of their lives [5]. This is exemplified through, for example, medical rationing and a lack of accessible information [4]. Disabled people are asked to resort to self-protection, *shielding* from COVID-19 – often without access to resources to support this – a practice which individualises the responsibility for protection against COVID-19, shifting it away from the state to individual actors [6]. Even when such shielding is possible, it has unintended consequences, ranging from the loss of informal-yet-necessary social support networks, to loneliness and food insecurity, exacerbating pre-existing inequities [7]. This discourse of *responsibilisation* fails to recognise that social privileges – in the form of social capital, education, and income – are not equally distributed among the population, a fact which has become even more evident in the current context [4].

The failures of this discourse have now been well-documented. In England and Wales, two-thirds of people who died from COVID-19 lived with disability, and it is estimated that disabled people had up to 11 times higher odds of dying from COVID-19 in the first two months of the pandemic compared to non-disabled people [8]. Some of the first contagion clusters emerged in care homes, turning into sensationalist headlines. In New York State, a recent study showed how people with intellectual and developmental disability living in residential group homes were at a greater risk of poor COVID-19 outcomes [9]. Lack of self-isolation and testing intersected with neglect and abandonment, leading to excess death rates in residential care homes. This is the result of government policies that failed to protect people, and are not a natural consequence of an inherent ‘vulnerability’. As documented in Australia, personal care workers may themselves work in precarious circumstances, placing them at higher risk of COVID-19 infection [10]. Low pay, insecure and casualised employment, and a poorly regulated sector meant that care workers can neither afford to stay at home if infected nor work in only one workplace. This, combined with low use and insufficient training related to personal protective equipment, repeatedly exposes people with disabilities – recipients of intimate or close care – to the risk of infection.

**Figure:**
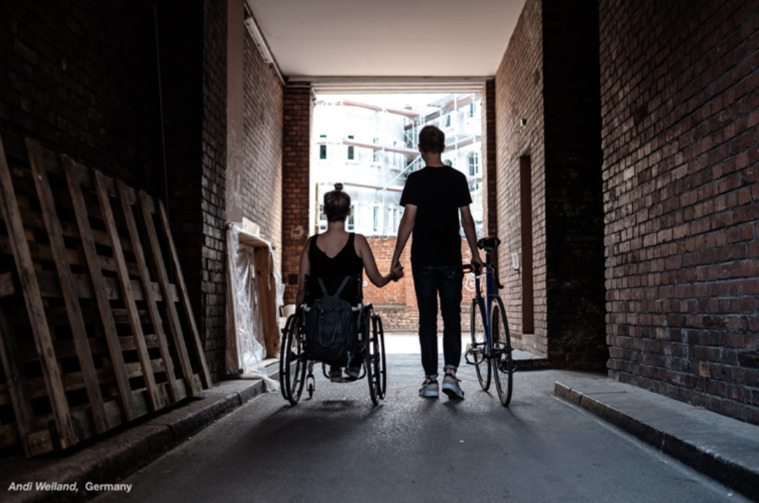
Photo: Final selected photographs for “Images of ability”; UN Enable Photo Exhibition for IDP.

The intersections of disability with poverty mean that disabled people often live in precarious situations, where it is difficult to maintain physical distancing and personal hygiene, often with poor access to clean water [1]. This renders people with disability more likely to contract and become seriously ill due to COVID-19. Being less likely to find work in the formal sector means that disabled people have less access to employment-based social security and health insurance, and reduced economic resilience in the current context. A recent UK survey found that 60% of disabled people reported problems accessing food, medicine, and other necessities [11]. This is unacceptable at the best of times, but can be lethal during a pandemic.

Attitudinal, environmental, and institutional barriers are even more pronounced in the case of people experiencing multiple forms of structural disadvantage, such as disabled women, children, and older people, disabled prisoners, homeless disabled people, and disabled people in situations of risk and humanitarian emergencies. These challenges are exacerbated by a general lack of accessible information, which is crucial for information dissemination throughout the response and recovery phases. For example, not all states provided sign language interpretation during the COVID-19 government briefings.

Governments across the world have introduced measures to protect the rights and well-being of disabled people and mitigate COVID-19 impacts in various areas, such as health, education, and housing. Disability-inclusive measures can be either direct – focused on ensuring the well-being of disabled people – or indirect – aimed at protecting the general population, and therefore, disabled people too – from the impact of the pandemic. A direct measure, for example, is the online provision of disability registration, which in many countries is linked to the provision of social and health care benefits, while an indirect measure is financial support for people of low socioeconomic status. A key component for the successful implementation of these measures is the availability of relevant and accessible information – such as sign language interpretation, easy-read information, and published recommendations specifically for disabled people – that ensures all necessary information regarding COVID-19 reaches them [12]. A study of four countries in South America highlighted the inadequacy of measures to fully protect the rights of disabled people and consider their needs [3].

In May 2020, 138 member states and observers issued a joint statement on the UN Secretary-General’s call for a disability-inclusive response to COVID-19, where they stated the need for “cooperation, investment and direct support from all stakeholders, including governments, the UN System, humanitarian actors, civil society, and representative organizations of persons with disabilities, as well as the private sector..[so as to promote] disability-inclusive local, national, and global responses” [13]. While the expression of a willingness for a disability-inclusive COVID-19 response is welcome, what is needed is urgent action. To date, government responses to disability inclusiveness in measures undertaken to address COVID-19 have been uneven and mostly disappointing, particularly considering that 182 states have ratified the UN Convention on the Rights of Persons with Disabilities (CRPD). Few countries have adopted comprehensive legislation that includes explicit protection of the rights of disabled people, while many have introduced measures that do not take into account the needs of disabled people. The UN Special Rapporteur on the rights of persons with disabilities stated that disabled people are often still not considered in government responses to the pandemic, resulting in measures that might inadvertently exclude them [13].

The extent to which disability-inclusive measures have been considered in the current pandemic—which itself poses an existential risk – requires examination. Urgent action is needed, ensuring the following [2,5,12]:

- Provision of accessible information;
- Provision of health services via telemedicine and through community-based networks, ensuring equitable healthcare access;
- Guidelines prohibiting blanket decisions on medical rationing solely on the grounds of disability;
- Employment and financial protection delivered through disability-related welfare provision;
- Development of support frameworks for people who need to shield from COVID-19 but who are outside of the social welfare or social care context (for example, reasonable adjustments in employment working arrangements);
- Education interventions and reasonable accommodations through online special education classes, accessible education activities, and distribution of educational materials;
- Social care services, including psychosocial support, personal assistance, and support for independent living;
- Prevention from and response to violence, in the forms of accessible hotlines for gender-based violence, especially for disabled women, and emergency services and shelters prepared to meet the needs of disabled people;
- Measures addressing the intersectional disadvantage disabled people face, including early release for disabled prisoners, and provision of accessible health services for homeless people; and
- Inclusion of disabled people in the recovery phase, ensuring that structural changes are implemented making societies more inclusive.

COVID-19 has exposed and amplified existing inequalities, leaving disabled people feeling excluded, discriminated against, and marginalised. As the pandemic unfolds, it is crucial that disabled people and their representative organisations are involved in decision-making processes, in line with Article 4(3) of the CRPD, with regards to both direct and indirect measures in the fight against COVID-19. Prevention of discrimination of disabled people and mitigation of the impacts of the pandemic for this population requires active commitment by key stakeholders to develop and implement effective disability-inclusive policies.
